# Effect of family "upward" intergenerational support on the health of rural elderly in China: Evidence from Chinese Longitudinal Healthy Longevity Survey

**DOI:** 10.1371/journal.pone.0253131

**Published:** 2021-06-18

**Authors:** Zhan Shu, Jinguang Xiao, Xianhua Dai, Yu Han, Yingli Liu

**Affiliations:** 1 School of Public Administration, Central China Normal University, Wuhan, Hubei Province, P.R.China; 2 Center for Labor and Social Security, Central China Normal University, Wuhan, Hubei Province, P.R.China; University of West London, UNITED KINGDOM

## Abstract

As health challenging rural elderly in an aging population, more attention is being paid on impact of family intergenerational support on the health of the elderly. This paper investigates the effects of children’s intergenerational economic support and non-economic support on physical, mental, and functional health of rural elderly in China in the mean while. This paper applies the 2014 Chinese Longitudinal Healthy Longevity Survey (CLHLS), in particular, applying exploratory factor analysis to ascertain latent variables and Structural Equation Model (SEM), and analyzes the impacts of "Upward" intergenerational support on health of rural elderly. As resulted, after controlling the socioeconomic status of the rural elderly, the family “upward” intergenerational support influences the elderly’s physical health at a percentage of 11.7%, mental health 29.8%, and physiological function 12.6%. Moreover, "Upward" economic support has a positive effect on physiological function (P<0.05). "Upward" non-economic support has negative effects on physiological function and mental health (P<0.05), while it has a positive effect on physical health. In addition, economically independent rural elderly are more likely to benefit from the health of "upward" intergenerational support, especially mental health. In particular, those results are robust. "Upward" intergenerational support plays an important role for the health of rural elderly. For the rural elderly of economic independence, to improve the quality of care and spiritual support, it is important to solve the health problems. In addition, it is necessary to build a comprehensive old-age security and support system for family, community, and society jointly to improve the health of the rural elderly.

## 1. Introduction

The depth and breadth of population ageing in China is unprecedented, and has become an important factor affecting the sustainable development of society and the realization of human health and well-being. As formulated by the Central Committee of China in the 14th five-year Plan for national development, the nation actively responses to the aging of the population. As the largest “healthy and vulnerable” group in China, the elderly has received extensive attention for their health. Under the dual urban-rural structure in China, there are differences in the old-age pension models of urban and rural. Community and social old-age pensions have been developing rapidly in cities, rural areas still continue the traditional "family" mode of elderly care,which relies mainly on individuals, families, or family. What are the respective roles of the family, the community and society in coping with the ageing process? What is the impact of the family-centered mode of old age on the elderly health and the mechanisms of influence? They are key issues to investigate. As early as the late 20th century, the relationship between family intergenerational relationship and elderly health was actively studied at home and abroad, but they do not reach final conclusion. For example, intergenerational support has a positive effect on the health of older people. As a kind of informal social support, intergenerational support can reduce the stress of old age and thus have a positive impact on health [1]. Children’s communication with their parents in the process of providing care and emotional support can bring good mental state to the elderly, which is conducive to health [2,3]. Older Chinese, especially women, are able to take care of themselves since they provide more grandchild care and housework than older men [4]. Thus, family relations are related to the health status and mortality of the elderly [5]. Intimate intergenerational relationships are more likely to reduce the risk of death of the elderly [6], and when family intergenerational relationship conflicts are more prominent, the elderly in poor family relationships are more likely to have poor self-evaluation of health and depression [7]. On the other hand, increased intergenerational support is beneficial to the health of older adults, a few have found the opposite. When the elderly perceive themselves in good physical condition, they tend not to need help from others (unless they are unable to take care of themselves). The photographs provided by their children may undermine the elderly’s evaluation of their own health and self-utility, leading to excessive dependence [8,9]. Children’s economic support not only increases the depression of rural elderly, but also has a negative impact on their life satisfaction [10,11].

As shown in the above literature, there is a relationship between family intergenerational support and the health of the elderly. The impact of intergenerational support was noticed on mental health, physiological function, but health is multi-dimensional, intergenerational support may have a positive impact on mental health, but also may easily ignore the negative impact on physiological function, and intergenerational support need be clearly divided, for instance, instrumental support, financial support, and emotional support [12,13]. Upon intergenerational support theories, for example, intergenerational solidarity model [14], feedback mode [15], family systems theory [16], filial piety theory [17], and altruism [18], this paper explores the relationship between kinds of children’s intergenerational support and health, which is directly related to the family support and the health of the elderly under the background of high aging in China.

Health is multidimensional, for example, Comprehensive Geriatric Assessment (CGA) [19], Older Americans Resources and Services (OARS) [20], PGCMAI [21], etc., and includes physical, mental and social well-being and not merely the absence of disease or infirmity [22–24]. These multidimensional health scales include the Activities of Daily Living (ADL), physical health status, mental health status, and other indicators [25–27]. It motivates this work to explore the relationship between intergenerational support and multidimensional health in a structural equation model.

In general, intergenerational support mainly passes downwards, i.e., from the old generation to the young generation, or at least a balanced state is reached between the two [28–31]. According to family ethics, the young people will provide care for their parents in the traditional ethical concept of “raising children for retirement safeguard”, while the parents will also provide care to their family to maintain family unity. Chinese families attach importance to the culture of filial piety [32]. With the acceleration of modernization, in spite of a recession of the filial piety culture in rural areas, the support offered by the young people is significant for the health and care of the elderly due to the relatively poor care and medical facilities for elders in rural areas compared to urban ones [33,34]. Therefore, most of the assistance and support received by the elderly in rural areas may come from their sons and daughters [35,36]. Hence, this work concentrates on the impact of “upward” family intergenerational support on the elderly people in terms of different healths in rural areas.

Previous literature explores the health problems of the elderly by one dimensional health index, for example, mental health. This work goes beyond those literature, and uses structural equation model and multidimensional health index [37]. In detail, this work applies multi-factor and multi-dimensional health, including the physical, psychological, and functional health in the mean time, and investigates the impact of different "upward" intergenerational support on those health of rural elderly.

## 2. Literature review

Intergenerational relationship is abstract, complex and composed of multiple dimensions [38,39], and represents geographical proximity, frequency and type of contact, level and form of communication [40]. In China’s rural areas, intergenerational support is closely related to the health of the elderly, for example, "upward" intergenerational support. Family support is indispensable for the elderly to receive care services and social support [41–43]. In particular, as a close-knit social unit, the family offers elderly care, financial support, and emotional support [44]. Most of the help and support for the rural elderly comes from the family [45]. The economic resources in the family are centered on the "upward" transfer [46] and affected by the number of children [47,48]. Intergenerational support includes economic support, the non-economic support, and emotional support [49–53].

Health, as an important component of human capital, is multidimensional [54]. Personal health is not merely the absence of disease, but also includes good mental, physical, behavioral, and social well-being [55,56]. In terms of physiological health, some literature take self-evaluation of health and objective physiological health status (BMI, chronic diseases) as indicattor [57,58]. Some take Activities of Daily Living (ADL) as a measure of physiological health [59]. Mental health not only refers to the opposite of mental disorders, but also emphasizes a state of positively facing life and self [60]. Different mental health indicators are adopted, for example, the individual’s depressive symptoms [61]. Those indicators include negative emotions such as loneliness, tension, anxiety, and interpersonal sensitivity [62–65]. Self-assessed mental health also measures mental health [66]. Good physical function is often reflected by an individual’s physical health, in which the body has no physiological disability, the manual function and the hearing and vision are better. Self-care ability and behavioral dysfunction in daily life indicates physical health. As the elderly’s daily behavioral function is lost, their physical health have problem and they depend on the assistance of others [67]. Both intergenerational family support and the health of the rural elderly have been analyzed [68–71]^.^ However, the impact of intergenerational family support are different on various health of the rural elderly. In particular, elderly in poor health would like to receive comprehensive support from their children [72]. This work divides health into mental health, physical health, and physiological function, and analyzes the influence of family "upward" intergenerational support on those health in the mean while.

Intergenerational economic support can not only solve the life problems, but also play a positive role in promoting the health and substitute to a certain extent for spiritual support for the elderly. Appropriately "upward" intergenerational financial support can reduce the incidence of elderly depression [73,74]. Children can improve the health, including mental health, and meet the various daily living and medical needs through economic support to the elderly [75,76]. Older adults, who get support from their children and who exchange financial and emotional support with them, are more likely to have higher life satisfaction [77]. However, once receiving more material support, the risk of depression increases significantly among the rural elderly, since the elderly feel uncomfortable [78,79]. Meanwhile, the financial support obtained has a negative impact on the life satisfaction of the elderly [80]. Children’s material support and assistance can improve the living standard which has an "energy-enhancing effect" on the physical health and self-care ability of the elderly [81]. So "up" intergenerational economic support has an impact on the elderly mental and physical health, but the impact of intergenerational support on one dimensional health does not meet the multidimensional health demands of rural elders in the mean time.

Intergenerational non-economic support also has an important impact on the health of the elderly. The traditional family care mode is the main channel to meet the demands of the elderly [82]. And it has a significant impact on the elderly’s physical and mental health [83]. Obtaining daily care has slowed the decline of the elderly’s physical health [84]. And older people with poor physical health and financial difficulties will rely more on the daily care provided by their children. Emotional comfort is more important when older people face health challenges such as physical deterioration [85]. At the same time, improving intergenerational emotional communication can promote the self-rated health of the elderly [86]. However, elderly people who receive more daily care will face greater health risks [87]. When children’s financial support is insufficient, moderate emotional support can play a substitute role, but excessive emotional support cannot achieve this effect [88], since children’s life care may undermine the elderly’s evaluation of their actual health status and self-utility. As analyzed, intergenerational support had no significant effect on self-rated health of the elderly [89], and the access to financial support or daily care accelerated the decline in self-care ability [90], not only due to abnormal body functions, but also because of external factors such as medical and health services and interpersonal relationships [91]. This paper applies intergenerational non-economic support, including emotional support, as non-economic support observation indicators, and Instrumental Activity of Daily Living (IADL), Activities of Daily Living (ADL), and physical function limitation as the observation indicators of the physical function to explore the impact of intergenerational non-economic support on the physical health, mental health, and physical function of rural elderly.

Though the impact of intergenerational support on the physical, mental, and self-rated health of the elderly was discussed, its impact on physiological functions, as we know, is kept silent. In addition, those literature ignore the multidimensional health in the mean time. Though rural elderly provide financial, care-giving, and emotional support to their children, children support their elder parents in most cases [92]. When elderly are at risk of health deterioration, adult child support is their main source of economic and non-economic help, and a close relationship remains between them for finance, care, and housework [93]. This paper investigates the influence of "upward" intergenerational support, economic and non-economic, on the physical health, mental health, and physical function of the rural elderly in the mean while.

## 3. Data resource and theoretical model

### 3.1. Data

The paper applies Chinese Longitudinal Healthy Longevity Survey (CLHLS) in 2014 and 2018 to discuss the impact of “upward” intergenerational support on the different health of rural elderly. The survey was carrieded out by Peking University and covered 23 provinces, autonomous regions, and municipalities, accounting for 85 percent of China’s total population. After data coding and assignment, the elderly who live in the rural area are selected, that is, the rural elderly, excluding samples less than 60 years old, and the final total sample size is 1407.

### 3.2. Variables

#### 3.2.1 The index selection of multidimensional health

The health of the elderly is multi-dimensional and is impossible to include all health indicators when modeling. This paper measures different health of the elderly from the following aspects:

First, physical health indicators consist of two-week prevalence and medical costs. The questionnaire asks, "How much did your inpatient and hospital stay cost in the past year? What is the prevalence of the elderly for two weeks?". Elderly people is in poor physical health if he/she is sick within two weeks and the medical expenses are higher.

Second, mental health is measured by the following indicators: "Do you often feel lonely?”, “Do you often feel nervous or afraid?”,”Do you think that the older you get, the less useful it is?”, “Do you feel as happy as when you were young?”, “Do you like to keep things clean and tidy?". After coding, the higher the weighted sum is, the poorer the mental health for elderly is.

Third, physiological functions. Functional limitations mainly include basic daily life activities, instrumental production and living activities, and advanced daily life abilities such as social contact and work [94]. Activities of Daily Living (ADL) includes six indicators. “Do you need help from others when you take a shower, do you need help from others when you dress, do you need help from others when you use the toilet, do you need help from others when you are indoors, can you control your bowel movements, and do you eat?” After coding (1 for “without assistance”, 2 for “one part assistance”, and 3 for “more than one part assistance”), the larger the total score of the 6 indexes by weighted sum is, the poorer the daily life ability of physiological function is. Instrumental Activity of Daily Living (IADL) includes “Can I go to my neighbor’s house? Can I go out and buy something by myself? Can I cook by myself? Can I wash my clothes by myself? Can I walk 2 miles in a row? Can I lift about 10 kg? Can you squat down and stand up three times in a row? Can you travel by public transportation alone? Can you lift a 5 kg weight?". After coding (1 for “yes”, 2 for “a little difficult”, and 3 for “unable to do so”), the larger the total score of the 8 indicators by weighted sum is, the poorer the Instrumental Activity of Daily Living is. Physical limitations include "Do you sleep well (1 for “very good”, 2 for “good”, 3 for “so so”, 4 for “bad”, and 5 for “very bad”)? Do you see if the circle has an opening with no glasses (1 for “can see and distinguish”, 2 for “can see only”, and and 3 for “can’t see”, 4 for “blind”)? Whether the hand can touch the neck (1 for “both hands”, 2 for “left hand/right hand”, and 3 for “neither hand”)? Whether the hand can touch the back (1 for “both hands”, 2 for “left hand/right hand”, and 3 for “neither hand”)? Can you raise your arm (1 for “two arms”, 2 for “only left arm/right arm”, and 3 for “neither left nor right arms”)? Can you stand up by yourself (1for “yes, without using hands”, 2 for “yes, using hands”, and 3 for “no”)? Do you have a hunchback (1 for “no”, 2 for “yes”)? Do you have difficulty hearing (1 for “no”, and 2 for “yes”)? Can you pick up the books on the ground (1 for “yes, standing”, 2 for “yes, sitting”, and 3 for “no”)? How many steps do you take in one rotation (0–30 steps)?" The larger the total score of the 10 indicators by weighted sum is, the poorer the Function health is.

#### 3.2.2 The index selection of explanatory latent variable

Parents and children are generally the same in terms of mutual support and assistance [95]. However, the two may be different in nature [96]. To better investigate the impact of family "upward" intergenerational support on the multidimensional health of the elderly in rural areas, the support from grandchildren was also included in the "upward" support for analysis. The independent variables are “upward” economic support and non-economic support (time care and spiritual comfort). The “upward” economic and non-economic support questionnaire in the database are "How much money (including cash and value of materials) did you get last year from your children and their spouses both living and not living with you?", "Who do you chat with most? If you have a thought or an idea, to whom do you speak first?", "What time support do your children and grandchildren provide when you need help with your daily activities?", "If you encounter problems or difficulties, whom to turn to first?". Variable description and statistics are shown in Table 1.

**Table 1:** Variable description and statistics (N = 1407).

Variables	Assignment	Minimum	Maximum	Mean	Standard deviation
**Physical Health(PH)**					
What is the prevalence of the elderly for two weeks?(E1)	0 = No 1 = Yes	0	1	0.20	0.40
How much did your inpatient cost in the past year? (E2)	——	0	99998	2638.21	8440.87
How much did your outpatient service charge in the past year? (E3)	——	0	99998	1470.22	4648.13
**Mental Health(MH)**					
Do you think that the older you get, the less useful it is? (E4)	1 = never 2 = seldom 3 = sometimes 4 = often 5 = always	1	5	2.91	1.13
Do you often feel lonely? (E5)	1 = never 2 = seldom 3 = sometimes 4 = often 5 = always	1	5	2.05	0.97
Do you often feel nervous and scared?(E6)	1 = never 2 = seldom 3 = sometimes 4 = often 5 = always	1	5	1.93	0.88
Do you like to make things clean and tidy? (E7)	1 = always 2 = often 3 = sometimes 4 = seldom 5 = never	1	5	2.28	0.77
Do you feel as happy as when you were young? (E8)	1 = always 2 = often 3 = sometimes 4 = seldom 5 = never	1	5	2.64	1.34
**Physiological Function** **(PF)**					
Instrumental Activity of Daily Living (IADL) (E9)	——	8	24	12.18	5.11
Activities of Daily Living (ADL) (E10)	——	6	17	6.55	1.63
Condition of limited physical function (E11)	——	10	46	21.01	7.53
**Intergenerational Economic Support(EC)**					
Grandchildren financial support (E12)	——	0	65000	494.45	2084.86
Daughter financial support (E13)	——	0	30000	1118.46	2170.52
Son financial support (E14)	——	0	80000	1560.92	4001.15
**Intergenerational Non-economic Support (INE)**					
If you encounter problems or difficulties, whom to turn to first?(E15)	0 = other 1 = child or grandchild	0	1	0.69	0.46
If you have a thought or an idea, to whom do you speak first?(E16)	0 = other 1 = child or grandchild	0	1	0.54	0.50
Who do you chat with most?(E17)	0 = other 1 = child or grandchild	0	1	0.43	0.50
What time support do your children and grandchildren provide when you need help with your daily activities?(E18)	——	0	168	17.74	27.76
**Socioeconomic Status(SS)**					
Living standard(E19)	1 = Poverty 2 = General 3 = Wealthy	1	3	2.	0.54
Last year’s household income(E20)	——	300	100000	27528.46	26917.95
Whether all the sources of living are adequate? (E21)	0 = not enough 1 = enough	0	1	0.78	0.42
**Features**					
Do you have any old-age security?	0 = No 1 = Yes (retirement pension or public old-age insurance)	0	1	0.35	0.48
Gender	0 = Male; 1 = Female	0	1	0.49	0.50
Age	——	67	114	83.66	9.32
years of schooling	——	0	18	2.29	3.21
smoke or not at present?	0 = no; 1 = yes	0	1	0.2	0.39
drink or not at present?	0 = no; 1 = yes	0	1	0.18	0.38
how often eat vegetables?	0 = seldom; 1 = quite often	0	1	0.88	0.31
how often eat fresh fruit?	0 = seldom; 1 = quite often	0	1	0.39	0.49

### 3.3 Basic model

The structural equation model is used to analyze the impact of different intergenerational support on the multidimensional health of rural elderly. It is composed of structural model and measurement model. The structural model represents the relationship between latent variables, while the measurement model describes the relationship between latent variables and observe variables. According to the previous literature, in the structural equation model constructed, the control variables as explicit variables can not be brought into the model, and the relationship between latent variables should be focused on [97–100]. More variables will be introduced to the model when the robustness test is carried out. The work will extract 6 potential variables (EC, INE, PH, MH, PF, SS), which involve 6 measurement models. In this paper, 3 observation indicators measure EC latent variables and 3 observation indicators measure PF latent variables. The measurement model is written as follows (other measurement models are not shown):

$$X_{1}=\lambda_{1\xi 1}+\delta_{1};X_{2}=\lambda_{2\xi 2}+\delta_{2};X_{3}=\lambda_{3\xi 3}+\delta_{3}$$

$$Y_{1}=\lambda_{1}\eta_{1}+\varepsilon_{1};Y_{2}=\lambda_{2}\eta_{2}+\varepsilon_{2};Y_{3}=\lambda_{3}\eta_{3}+\varepsilon_{3}$$

The above regression equations can be expressed by matrix equations, for example, (2) and (3), Let ξ and η respectively represent exogenous latent variables, for example, EC, and endogenous latent variables, for example, different health dimensions. For simple notation, we use vector and matrix to represent the whole model as follows.

$$\eta =B\eta +\Gamma \xi +\zeta =>[\begin{array}{l} \eta_{1}\\ \eta_{2}\\ \eta_{3} \end{array}]=[\begin{array}{l} \beta_{11}\beta_{12}\beta_{13}\\ \beta_{21}\beta_{22}\beta_{23}\\ \beta_{31}\beta_{32}\beta_{33} \end{array}][\begin{array}{l} \eta_{1}\\ \eta_{2}\\ \eta_{3} \end{array}]+[\begin{array}{l} \gamma_{11}\gamma_{12}\gamma_{13}\\ \gamma_{21}\gamma_{22}\gamma_{23}\\ \gamma_{31}\gamma_{32}\gamma_{33} \end{array}][\begin{array}{c} \xi_{1}\\ \xi_{2}\\ \xi_{3} \end{array}]+[\begin{array}{c} \zeta_{1}\\ \zeta_{2}\\ \zeta_{3} \end{array}]$$ (1)

where η represents the three latent variables explained in this paper: η_1_ is PH, η_2_ is MH, and η_3_ is PF. ξ represents three explanatory latent variable: ξ_1_ is EC, ξ_2_ is INE, and ξ_3_ is the SS of the elderly. ***B*** represents the structural coefficient matrix of the relationship among the three explained latent variables, η_1_, η_2_, and η_3_. ***Γ*** represents the structural coefficient matrix of the relationship between the explained latent variable and the explanatory latent variable. And ζ is the prediction error of the structural model.

The measurement model of the explained latent variable η is shown in Eq (2). Three observation indicators measure η_1_ (PH): Y_1_ is the outpatient cost (E_3_), Y_2_ is the inpatient cost (E_2_), and Y_3_ is the two-week disease status (E_1_). Other observed indicators of explained latent variables have been mentioned above. Λ_y_ is the factor load between the observed indicators (y_1_~y_11,_ E_1_-E_11_) and the explained latent variables (η_1_, η_2_, η_3_). And ε is the measurement error of the explained latent variables.


$$Y=\Lambda_{Y}\eta +\varepsilon$$ (2)

The measurement model of explanatory latent variable ξ is shown in Eq (3). Three observation indicators measure “upward” economic support ξ_1_: x_1_ is the economic support of the son (E_12_), x_2_ is the economic support of the daughter (E_13_), and x_3_ is the economic support of the grandchildren (E_14_). Other observed indicators of explanatory latent variable have been mentioned above. Λ_**x**_ is the factor load between the observed indicators (x_1_~x_10,_,E_12_-E_21_) and the explanatory latent variable (ξ_1_, ξ_2_, ξ_3_). And δ is the measurement error of explanatory latent variable.


$$X=\Lambda_{X}\xi +\delta$$ (3)

In this paper, AMOS is used to analyze the impact of different intergenerational support on different health. Compared with traditional methods, the structural equation model integrates measurement and analysis into one, and at the same time estimates the measurement indexes and latent variables in the model. The model not only solves both health and intergenerational support as multi-dimension, but also clarifies the relationship between of them. In addition, the data meets the requirements to formulate the model, maximum likelihood estimation is carried out for parameter estimation, and kurtosis and skewness are less than 3 and 8. Using exploratory factors to extract 6 latent variables, the KMO value is greater than 0.7, so it is suit for factor analysis. The 6 latent variables are meaningful for the measured variables.

## 4. Results

### 4.1 Estimation results of structural equation model

**Fig 1:**
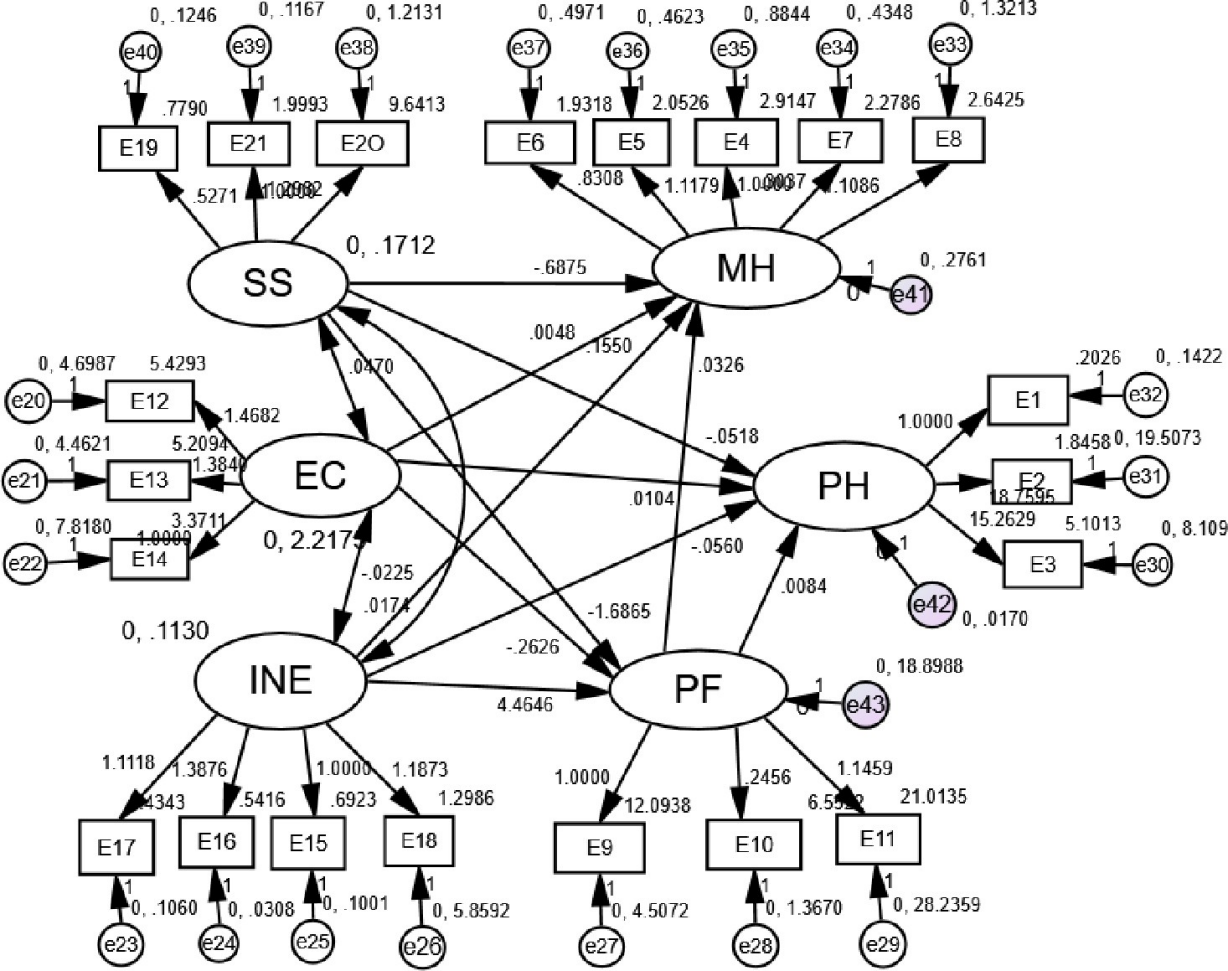
Structural equation model of the effect of intergenerational support on the health of the elderly in rural areas and its mechanism.

To intuitively understand the impact of intergenerational support on the health of rural elderly, the maximum likelihood estimation is carried out. The standardized of path coefficients, variable residuals, and factor loads are shown in Fig 1 (normalized coefficients).

First and foremost, as seen from Table 2, intergenerational support has a significant impact on the physical health of rural elderly (P<0.05). Specifically, intergenerational economic support has a negative impact on the physical health of the elderly, but intergenerational non-economic support has a positive impact on physical health. That is, the more non-economic support the elderly receive from their children or grandchildren, the less possibility for them to be ill and cause medical expenditure. So moderate care support and mental comfort positively improve physiological health, since most of elderly in this sample are at high age, the children’s non-economic support to some extent reduces the cost of manual care and the total medical expenses for the elderly. In addition, physical health includes medical service demand and utilization, hence it is not yet possible to judge whether the impact of physiological health is upon the impact on health demand or the use of health services.

**Table 2:** Parameter estimation for structural equation model (N = 1407).

Model	Variable relationship			Estimate	S.E.	C.R.	P	R^2^
Structural Equation Model	PH	<---	EC	0.01	0.004	2.398	0.016	0.117
	PH	<---	INE	-0.056	0.019	-3.004	0.003	
	PH	<---	SS	-0.052	0.018	-2.907	0.004	
	PH	<---	PF	0.008	0.002	4.917	0.000	
	MH	<---	EC	0.005	0.015	0319	0.75	0.298
	MH	<---	INE	0.155	0.065	2.402	0.016	
	MH	<---	SS	-0.687	0.082	-8.339	0.000	
	MH	<---	PF	0.033	0.005	6.34	0.000	
	PF	<---	EC	-0.263	0.105	-2.492	0.013	0.126
	PF	<---	INE	4.465	0.432	10.341	0.000	
	PF	<---	SS	-1.686	0.391	-4.31	0.000	
Measurement Equation model	E1	<---	PH	1				0.119
	E2	<---	PH	18.76	2.568	7.305	0.000	0.258
	E3	<---	PH	15.263	2.302	6.629	0.000	0.357
	E4	<---	MH	1				0.308
	E5	<---	MH	1.118	0.074	15.168	0.000	0.515
	E6	<---	MH	0.831	0.059	14182	0.000	0.353
	E7	<---	MH	0.304	0.036	8.383	0.000	0.077
	E8	<---	MH	1.109	0.08	13.837	0.000	0.268
	E9	<---	PF	1				0.828
	E10	<---	PF	0.246	0.01	25.294	0.000	0.488
	E11	<---	PF	1.146	0.047	24.612	0.000	0.501
	E12	<---	EC	1				0.221
	E13	<---	EC	1.384	0.11	12.589	0.000	0.488
	E14	<---	EC	1.468	0.121	12.174	0.000	0.504
	E15	<---	INE	1				0.53
	E16	<---	INE	1.388	0.047	39.827	0.000	0.876
	E17	<---	INE	1.112	0.041	27.23	0.000	0.568
	E18	<---	INE	1.187	0.205	5.8	0.000	0.026
	E21	<---	SS	1				0.595
	E20	<---	SS	1.298	0.112	11.593	0.000	0.192
	E21	<---	SS	0.527	0.044	11.975	0.000	0.276
Model fitting index	fitting index	χ2/df	CFI	TLI	NFI	IFI	RFI	RMSEA
	standards	<5	>0.9	>0.9	>0.9	>0.9	>0.9	<0.05
	Model results	3.644	0.929	0.915	0.905	0.93	0.886	0.043

Besides, intergenerational non-economic support has a significant negative effect on the mental health (P<0.05). Intergenerational economic support has no significant effect on the mental health (P = 0.75). Some studies believe that moderate emotional support play a substitute role when children’s economic support is insufficient, but excessive emotional support does not produce consistent results. The previous study believed that the internal support of the family had a positive impact on the health of the elderly upon the demand of the elderly. However, in rural areas, when the capacity of intergenerational non-economic care support is insufficient, or the willingness of support is low, and external support is lack, it is difficult to effectively meet the health demand of rural elderly.

Last but not least, intergenerational economic support is indispensable to solve the health problems of the elderly in rural areas, and the ability of family care support also needs to be further improved. As earlier studies shown, after entering old age, Chinese residents not only have the characteristics of early illness and long duration of illness, but also often face the risk of disability and partial disability, and their health is not optimistic, but these results are not entirely due to Human aging or natural phenomena. As Table 2 revealed, intergenerational economic support has a positive effect on the elderly’s physiological function(P<0.05). The more economic support, the lower the possibility of the elderly losing their physiological function. At the same time, non-economic support has a negative impact on physiological function. Lack as medical resources are in rural areas, when the intergenerational non-economic support has a negative impact on the physiological function of the elderly, children fail to provide support according to scientific methods does not necessarily improve the physiological function for elderly. Lack as children’s caring ability or willingness to support and external support, it is difficult to effectively meet the health demand of the elderly in rural areas. The more supportive care children and grandchildren provide, the less conducive to the improvement of the elderly’s physiological function.

In this paper, the model is acceptable after considering the degree of freedom, that is, the NC value is 3.644<5. However, for other goodness-of-fit indices [101,102], CFI = 0.929, TLI = 915, NFI = 905, IFI = 0.93, RFI = 886, and RMSEA = 0.043<0.05. The single equation R^2^ of the impact of the three explanatory latent variables on the physical health, mental health, and physiological function of the rural elderly is 0.117, 0.298, and 0.126, respectively. So the impact of different intergenerational support on the different health of rural elderly is different. After controlling the social and economic status of the elderly, the family “upward” intergenerational support influence the elderly’s physical health at a percentage of 11.7%, mental health 29.8%, and physiological function 12.6%.

### 4.2 Heterogeneity analysis and robustness test of structural equation model

#### 4.2.1 Heterogeneity analysis

This section answers following questions: Do the impacts of offspring intergenerational support on the health of the elderly have significant differences within different gender and pension security? What can policy implications be drawn if differences do exist?

In terms of gender, intergenerational economic support exerts a positive effect on the physiological functions of elderly females. Intergenerational non-economic support exerts a positive effect on the physiological health of elderly females, but a negative effect on their physiological functions. In contrast, intergenerational non-economic support exerts a positive effect on the physiological health of elderly males. Hence, the health of elderly females is more susceptible to offspring’s intergenerational support. As the data of sixth national population census of China revealed, there is huge contrast in gender ratio with females accounting for approximately 2/3 [103]. To a certain extent, the future aged society is basically an aged female society, which not only affects the overall health of the elderly, but also imposes greater challenge to the family, society, and government in terms of elderly care, medical treatment, and old-age security [104,105]. “Males managing external affairs while females managing internal affairs" has long been the traditional pattern of labor division in rural areas. Compared with males, females are tasked with burdensome agricultural labors, on top of which tremendous housework is exerted. Pressures, coupled with burdensome labor, are detrimental to female health [106]. Despite the widespread opinion that elderly females have longer lifespans than males, they are likely to be in worse health condition in their late stage of life [107]. What’s worse, elderly female are suffering from higher rates of chronic diseases than elderly males, and sinking in a state of lack of social interactions [108,109].Thre are gender difference in terms of health status for the elderly aged at 83.66 on average, in particular, the elderly females are suffering from poorer health than elderly males.

As the negative effect of offspring’s non-economic support on the physiological function of the elderly indicated, it is unfeasible to refine the health of the elderly in rural areas by solely relying on offspring’s intergenerational support. The specific results are shown in Table 3.

**Table 3:** Heterogeneity analysis.

Variable relationship			Female	Male	Without pension security	With pension security
PH	<---	EC	0.01 (0.007)	0.007 (0.006)	0.015** (0.006)	0.004 (0.007)
PH	<---	INE	-0.061* (0.03)	-0.022 (0.027)	-0.015 (0.022)	0-.079* (0.038)
PH	<---	SS	-0.051 (0.026)	-0.041 (0.025)	-0.066** (0.023)	-0.033 (0.028)
PH	<---	PF	0.009** (0.003)	0.012*** (0.003)	0.01*** (0.002)	0.008** (0.003)
PH	<---	education	0.017 (0.025)	0.01 (0.024)	-0.006 (0.02)	0.016 (0.03)
PH	<---	age	-0.066** (0.023)	-0.035* (0.018)	-0.049** (0.016)	-0.035 (0.022)
PH	<---	Vegetables (proxy variable)	0.009 (0.024)	0.012 (0.024)	0.006 (0.02)	0.014 (0.026)
MH	<---	EC	0.003 (0.021)	-0.01 (0.022)	0.046* (0.02)	0-.067* (0.027)
MH	<---	INE	0.148 (0.093)	0.181 (0.1)	0.216** (0.082)	0.086 (0.119)
MH	<---	SS	-0.625*** (0.11)	-0.666*** (0.115)	-0.688** (0.103)	-0.676*** (0.141)
MH	<---	PF	0.029*** (0.007)	0.046*** (0.008)	0.041*** (0.007)	0.026** (0.009)
MH	<---	Education	-0.102 (0.81)	-0.044 (0.086)	-0.122 (0.072)	-0.028 (0.101)
MH	<---	age	-0.167** (0.061)	-0.066 (0.061)	-0.142** (0.052)	-0.032 (0.079)
MH	<---	Vegetables (proxy variable)	0.159* (0.08)	-0.043 (0.084)	0.079 (0.071)	0.048 (0.096)
PF	<---	EC	-0.333* (0.151)	-0.031 (0.134)	0-.302* (0.133)	0.079 (0.172)
PF	<---	INE	2.508*** (0.634)	2.588*** (0.613)	3.707*** (0.536)	1.486 (0.769)
PF	<---	SS	-1.564** (0.528)	-1.953*** (0.52)	-1.892***(0.479)	-1.596* (0.643)
PF	<---	education	-0.012 (0.568)	-0.09 (0.536)	0.171 (0.492)	-0.424 (0.662)
PF	<---	age	3.240 (0.399)	2.459*** (0.365)	2.634*** (0.328)	3.379*** (0.485)
PF	<---	Vegetables (proxy variable)	-0.331 (0.553)	-0.450 (0.525)	-0.318 (0.486)	-0.466 (0.628)
Model fitting index			χ2/df = 2.345	χ2/df = 1.943	χ2/df = 2.658	χ2/df = 1.755
			CFI = 0.911	CFI = 0.936	CFI = 0.918	CFI = 0.927
			TLI = 0.892	TLI = 0.923	TLI = 0.901	TLI = 0.911
			NFI = 0.856	NFI = 0.878	NFI = 0.876	NFI = 0.847
			IFI = 0.912	IFI = 0.937	IFI = 0.919	IFI = 0.928
			RFI = 0.826	RFI = 0.853	RFI = 0.085	RFI = 0.816
			RMSEA = 0.045	RMSEA = 0.037	RMSEA = 0.043	RMSEA = 0.039

Note

* significant at P<0.05

** significant at P<0.01

*** significant at P<0.001.

As resulted in Table 3 among the elderly without pension security, intergenerational economic support has a positive impact on physiological functions and a negative impact on physical and mental health. Intergenerational non-economic support has a negative impact on both physical function and mental health. Intergenerational economic support has a positive effect on mental health for the rural elderly with pension security. Rural eldly with pension security do not rely too much on the family, but intergenerational support is more conducive to the improvement of their health. The accessibility of medical services and the level of community medical and elderly care services in rural areas are significantly worse than those in urban areas. Women, rural areas, and low-income groups are more likely to fall into long-term multidimensional health poverty. The health risks of the elderly without pension security are prominent [110]. Therefore, it is important to solve the rural elderly health problems by improving the rural security system and giving the elderly appropriate economic support to ensure the economic independence of the elderly, and increasing the willingness and quality of family non-economic support. According to the evaluation criteria in Table 3 and the analysis above, the effects of intergenerational support on the health of the elderly are significantly different due to the gender of the elderly and whether they had pension security or not.

#### 4.2.2 Robustness test

*4*.*2*.*2*.*1 Add variables to the basic model*. To test the robustness of the benchmark estimation results, this paper adds more variables into the original model. Added senior age (0 for “80 and below”, 1 for “80 and above”), education (0 for “primary school and below”, 1 for “primary school and above”), Do you smoke or not? (0 for “No”, 1 for “Yes”), Do you drink? (0 for “No”, 1 for “Yes”), Do you eat vegetables and fruits regularly (0 for “No”, 1 for “Yes”). S1 Table shows the estimated results of the age, education, and economic status of the rural elderly. The regression coefficient of children’s intergenerational economic support is significantly negative on physical health at the level of 5%, and is not statistically significant on mental health and physical function. The regression coefficients of intergenerational non-economic support are significantly positive on physical health at the level of 5%, and are significantly negative on mental health and physical function at the levels of 5% and 1%. Model is fitted well, sinceχ^2^/df = 3.432, CFI = 0.926, TLI = 0.911, NFI = 0.9, IFI = 0.927, and RMSEA = 0.042. The family "up" intergenerational support explains different health, for example, mental health (30.3%), physical function (20.7%), and physical health (13.9%). The direction and significance level of the regression coefficient are consistent with the empirical results of the benchmark model after adding the control variables, so the estimated results above are robust.

In S2 Table, the life style is added respectively in addition to age, education, and economic status of the rural elderly. The estimated results above are also robust.

*4*.*2*.*2*.*2 Adjust the model*. In the structural equation model, the correlation between the latent variables turns into causality, and the double arrow of the path turns into a pointed single arrow. In practice, if endogenous variables can be measured with multiple latent variables, exogenous explanatory variables are explicit variables, better results can be obtained compared with the regression model where explainable variables are explicit variables. This is a model of Multiple Indicators and Multiple Causes (MIMC), a kind of special structural equation model. A set of exogenous variables predicts one or more endogenous latent variables, where exogenous variables have no measurement error. The MIMC model of one endogenous latent variable runs as follows.


η=a+ΓX+ζ (4)

6 exogenous demonstrable variables are determined in this paper. Non-economic support is replaced by the care time provided by the children and the chatting situation between the children and their parents, and the financial support provided by the sons, daughters, and grandchildren is sumed up as the economic support provided by the children. Including the elderly’s self-assessment of living standard, education status, and whether they often eat vegetables (proxy variable) as covariates, a MIMC model of 3 endogenous latent variables is carried out, see S1 Fig. On the left side of the model is a group of exogenous obvious variables to explain the 3 endogenous latent variables (different health dimensions). On the right is the measurement model.

As shown in the model estimation, at first, the negative effect of children’s economic support on the physical health of the elderly is significant at 0.001 level, but the positive effect on the physical function is significant at 0.001 level, while the negative effect on the mental health is not significant. Second, chatting between children and the elderly had a significant positive effect on physical health at 0.001, but no significant effect on mental health, and a significant negative effect on physiological function at 0.001. Thirdly, the negative effect of child care time support on the physical health of the elderly is significant at 0.001 level, while the negative effect on the mental health is not significant, and the negative effect on the physical function is significant at 0.001 level. Physical health is evaluated by medical expenses and two-week illnesses. Accoding to the statistics, educational background of the elderly in rural China is much limited they are unfamiliar with the urban medical environment and have few communications with other people. The elderly, who have health problems and economic difficulties, rely on their children for daily care and financial support. Therefore, daily care doesn’t seem to benefit the physical health of the elderly. For MIMC model, CFI = 0.936, TLI = 917, NFI = 909, IFI = 0.937, and RMSEA = 0.038<0.05 which are higher than the standard, so the model is acceptable. The results are robust. The results in detail are shown in S3 Table.

*4*.*2*.*2*.*3 Adjust the sample*. According to the existing literature, the elderly in rural areas in China is indicated by rural household registration. However, to make this study more accurate, the rural elderly who born and live in rural areas with informal occupations before the age of 60 are sampled, and the elderly withdrawing from the city and living in rural areas after retirement are excluded. Moreover, observation indicators were screen by the exploratory factor analysis as same as the basic model for determining latent variables upon the database of 2018. Hence, the effect of intergenerational support of children of rural families on elderly’s multidimensional health is estimated with the SEM model.

As shown, all robust results are consistent with those of the basic model, except for the effect of children’s intergenerational supports on physical health of elderly in rural areas, since the data in 2018 are adopted to carry out the robust test with inpatient and outpatient medical expenses of the elderly in rural areas as observational indicators of physical health. During China’s 13th Five-Year Plan, a healthy China strategy has been proposed, in which measures should be taken to promote the lower levels of medical resources, and improve the rural medical service system. In particular, the health poverty alleviation should be leaned towards the rural areas. Hence, a work promotion mechanism dominated by the government and involved by all parts should be established for dealing with key tasks such as medical security for the impoverished people, optimization of medical services for the impoverished people, disease prevention and control in poverty-stricken areas, and improvement of medical service capabilities in accordance with the work path of security, treatment, and prevention. In this way, a comprehensive medical insurance system can be provided to improve the regional medical and health services for the impoverished people in poverty-stricken areas, so that people in financial difficulty can afford to see a doctor, treat the disease, and get sick less. Since medical expenses for the elderly in rural areas are secured, intergenerational economic support and non-economic support of children in rural families have no statistical significance on the physiological health of the elderly in rural areas. The results in detail are shown in S2 Fig and S4 Table.

## 5. Discussion

Family pension mode is a typical pension way in China, which guarantees the health and life of the elderly to the greatest extent [111]. But this mode makes a large part of young people bear huge economic and living burdens. And for the elderly, excessive intergenerational family dependence is also not conducive to improving their health. With the diversification of pension modes at hand, the improvement of community pension functions and the increase of pension institutions, diversified choices are possible for the elderly [112]. However, family pension is still the main pension mode for the rural elderly because of the weak social pension function in rural areas of China [113].

First of all, family “upward” intergenerational economic support has a positive impact on the physiological function of the rural elderly, but it is harmful to the physiological health. As the “inadequate” situation of children’s daily care, the children’s financial support is still crucial to rural elderly [114]. The positive effect of intergenerational economic support on the physiological function of the rural elderly may be attributed to the intensity increase of social support for the elderly, who can have more social support resources, thus leading to the improvement of physiological function. However, it can not be judged whether the negative impact of economic support on physiological health is upon the increase of health demand caused by the lack of care support or the excessive use of health services.

Secondly, intergenerational non-economic care support has a positive effect on physical health and a negative effect on physiological function and mental health. In Chinese filial piety culture, children need to bear the “support obligation” of their parents and provide appropriate economic and non-economic support [115]. Intergenerational non-economic support has an effect on reducing medical expenses for the elderly and improving physical health. The mental health will be better if the elderly have a good relationship with their caregivers in the family. However, given intergenerational support willingness and ability of children, non-economic support in rural families not only fails to improve the mental health status, but also exacerbates the loss of physical functions for elderly. Since family members are primary human resources of care and services for the elderly, in particular, incapacitated or of disease, informal care, services, and care are demanded.

Finally, economic independence of the elderly can optimize the health effect of intergenerational support. Since the New Rural Pension Insurance System was introduced in 2009, the economic independence of the insured elderly has been enhanced [116]. As indicated, the New Rural Pension Insurance squeezed out the "upward" intergenerational economic support and the "upward" time and service support [117]. Mutual endowment mode cannot adapt to the current rural situation [118], so mutually beneficial pension mode should be advocated, and service or the caregivers should be paid [119]. In particular, the elderly need appropriately maintain economic independence. The "upward" intergenerational support is not conducive to improve the mental health status of the rural elderly who lack pension security because they rely too much on family economic support. The family "upward" intergenerational support is more effective in improving the physical and mental health for the rural elderly who are relatively economic independent.

## 6. Conclusions

Intergenerational family economic support plays an important role in improving the elderly’s physiological function, but non-economic support does not have a positive impact on the elderly’s physiological function and mental health. Due to the lack of external support, the health security challenges the rural elderly more than urban elderly.

First and foremost, build a multi-integrated elderly care mode of family, community, and society. To improve rural community support and health services, the health status of the rural elderly, and corresponding intervention, a "positive aging" social and cultural environment need be built.

Besides, optimize the structure and improve the quality of intergenerational family support. In policy, we should aid the elderly family, and lessen the children’s burden of family support. We should meet the elderly health and pension demand, thus build up a peaceful family environment.

Last but not least, improve the rural pension and medical security system to promote the economic independence of the elderly. The economic dependence of rural elderly on their children not only leads to financial burden of their children, but also influences the elderly’s own health. At family level, it actively advocates the high-quality intergenerational relationship of "reciprocity, interaction, and mutual assistance", increases children’s willingness and level of intergenerational support, and promotes the independence of rural elderly. At national level, it develops institutions of elderly care and nursing for the elderly as a supplement to family support.

### Limitations

There are several limitations in our study. This paper mainly analyzes the impact of family "upward" intergenerational support on the health of rural elderly people (children’s support to parents), but does not analyze the impact of family "downward" intergenerational support on the health of rural elderly people (parents’ support to children), which may also have an impact on health. In addition, in this paper, the measurement of multidimensional health does not completely follow the existing literature, but similar indicators are chosen according to the dataset.

## Supporting information

S1 Fig Multiple Indicators and Multiple Causes (MIMC) model path diagram. (TIF)

S2 Fig The model path diagram of structural equation model of the effect of intergenerational support on the health of the elderly in rural areas and its mechanism (2018 data). (TIF)

S1 Table Add variables to the base model (age+education+ economic status). (DOCX)

S2 Table Add variables to the base model (age+education+economic status+lifestyle). (DOCX)

S3 Table Parameter estimation results for multiple indicators and multiple causes. (DOCX)

S4 Table Adjust the study sample (2018 data). (DOCX)
