# Supplementary figures and images for: Effect of family "upward" intergenerational support on the health of rural elderly in China: Evidence from Chinese Longitudinal Healthy Longevity Survey
Source: PLoS One. 2021 Jun 18;16(6):e0253131. doi: 10.1371/journal.pone.0253131 (PMC8213075; doi:10.1371/journal.pone.0253131)

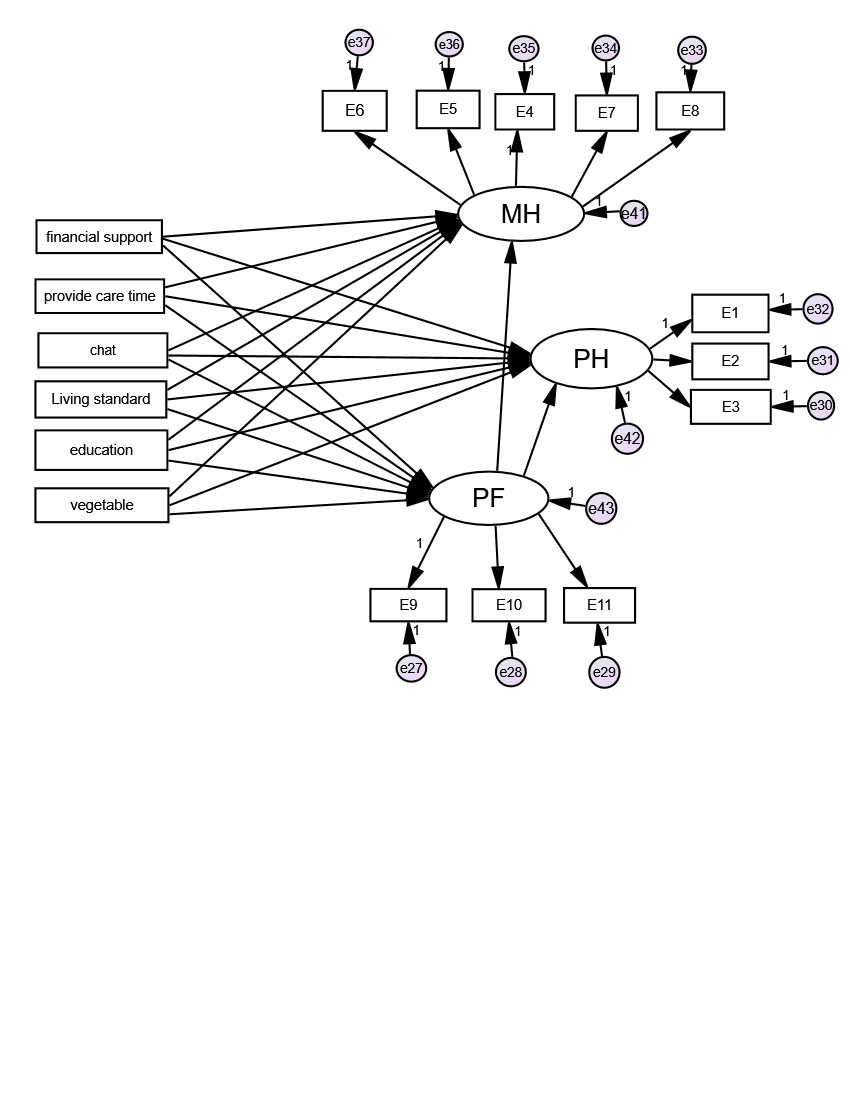

Supplement: S1 Fig — (TIF) [file pone.0253131.s001.tif]

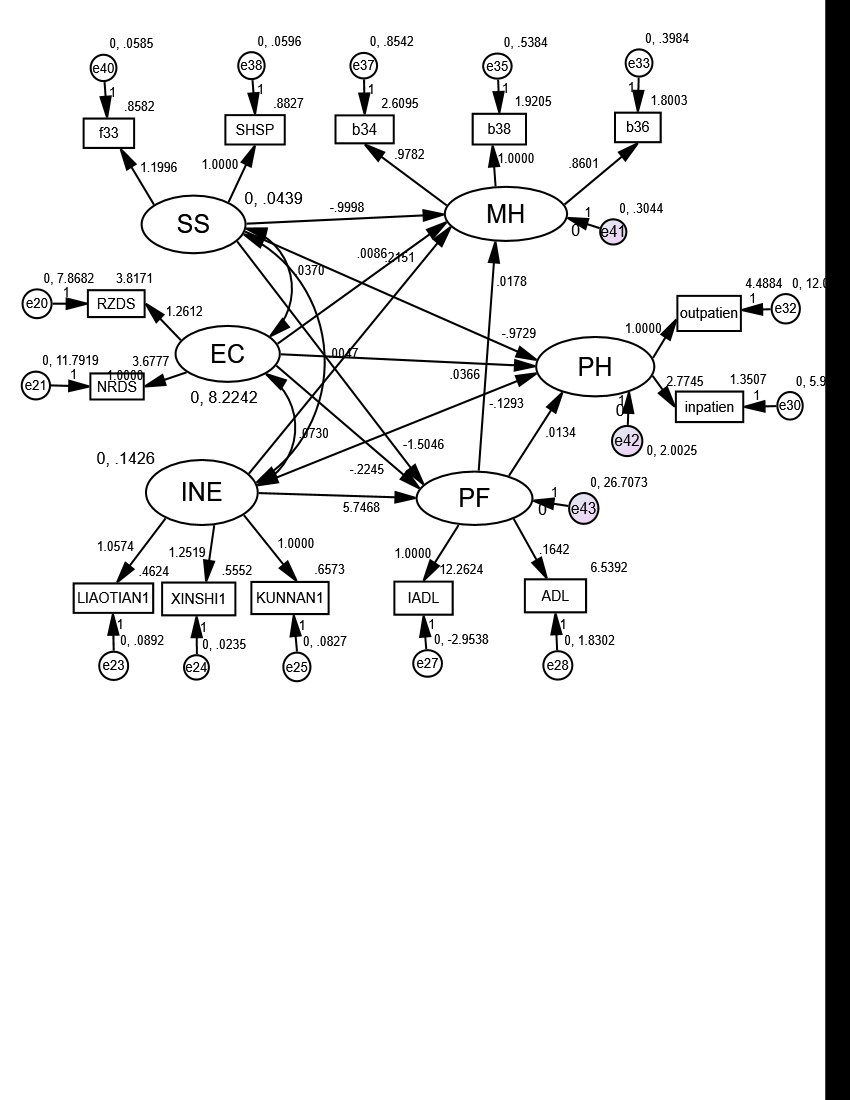

Supplement: S2 Fig — (TIF) [file pone.0253131.s002.tif]
