# Supplementary material for: Effect of family "upward" intergenerational support on the health of rural elderly in China: Evidence from Chinese Longitudinal Healthy Longevity Survey
Source: PLoS One. 2021 Jun 18;16(6):e0253131. doi: 10.1371/journal.pone.0253131 (PMC8213075; doi:10.1371/journal.pone.0253131)
Supplement: S1 Table — (DOCX) [file pone.0253131.s003.docx]

Add variables to the base model（age+education）（N=1407）

| **Model** | **Variable relationship** | | | | | | Estimate | | S.E. | | C.R. | | | P | R^2^ |  |
| --- | --- | --- | --- | --- | --- | --- | --- | --- | --- | --- | --- | --- | --- | --- | --- | --- |
| Structural Equation Model | PH | | <--- | | EC | | 0.009 | | 0.004 | | 2.152 | | | 0.031 | 0.139 |  |
|  | PH | | <--- | | INE | | -0.038 | | 0.019 | | -2.029 | | | 0.042 |  |  |
|  | PH | | <--- | | SS | | -0.047 | | 0.018 | | -2.678 | | | 0.007 |  |  |
|  | PH | | <--- | | PF | | 0.010 | | 0.002 | | 5.295 | | | *** |  |  |
|  | PH | | <--- | | age | | -0.049 | | 0.013 | | -3.683 | | | *** |  |  |
|  | PH | | <--- | | education | | 0.012 | | 0.016 | | 0.729 | | | 0.466 |  |  |
|  | PH | | <--- | | EC | | 0.001 | | 0.015 | | 0.095 | | | 0.924 | 0.303 |  |
|  | PH | | <--- | | INE | | 0.194 | | 0.067 | | 2.912 | | | 0.004 |  |  |
|  | PH | | <--- | | SS | | -0.674 | | 0.082 | | -8.234 | | | *** |  |  |
|  | PH | | <--- | | PF | | 0.036 | | 0.005 | | 6.615 | | | *** |  |  |
|  | PH | | <--- | | age | | -0.113 | | 0.043 | | -2.601 | | | 0.009 |  |  |
|  | PH | | <--- | | education | | -0.084 | | 0.059 | | -1.417 | | | 0.157 |  |  |
|  | PF | | <--- | | EC | | -0.170 | | 0.102 | | -1.665 | | | .096 | 0.207 |  |
|  | PF | | <--- | | INE | | 3.024 | | 0.432 | | 7.007 | | | *** |  |  |
|  | PF | | <--- | | SS | | -1.827 | | 0.378 | | -4.832 | | | *** |  |  |
|  | PF | | <--- | | age | | 2.886 | | 0.271 | | 10.633 | | | *** |  |  |
|  | PF | | <--- | | education | | -0.034 | | 0.394 | | -0.085 | | | 0.932 |  |  |
| Model fitting index | | fitting index | χ2 /df | | CFI | | TLI | | NFI | | IFI | | RFI | RMSEA | | |
|  |  | standards | <5 | | >0.9 | | >0.9 | | >0.9 | | >0.9 | | >0.9 | <0.05 | | |
|  |  | Model results | 3.432 | | 0.926 | | 0.911 | | 0.900 | | 0.927 | | 0.879 | 0.042 | | |

Note: *** significant at P<0.001.
