# Supplementary material for: Effect of family "upward" intergenerational support on the health of rural elderly in China: Evidence from Chinese Longitudinal Healthy Longevity Survey
Source: PLoS One. 2021 Jun 18;16(6):e0253131. doi: 10.1371/journal.pone.0253131 (PMC8213075; doi:10.1371/journal.pone.0253131)
Supplement: S2 Table — (DOCX) [file pone.0253131.s004.docx]

**Add variables to the base model（age+ education+ lifestyle）**

**1、age+ education+ lifestyle（drink）**

| **Model** | **Variable relationship** | | | | **Estimate** | **S.E.** | **C.R.** | **P** | **R^2^** |  |
| --- | --- | --- | --- | --- | --- | --- | --- | --- | --- | --- |
| Structural Equation Model | PH | <--- | EC | | 0.010 | 0.004 | 2.208 | 0.027 | 0.142 |  |
|  | PH | <--- | INE | | -0.037 | 0.019 | -1.998 | 0.046 |  |  |
|  | PH | <--- | SS | | -0.048 | 0.018 | -2.699 | 0.007 |  |  |
|  | PH | <--- | PH | | 0.010 | 0.002 | 5.296 | *** |  |  |
|  | PH | <--- | education | | 0.010 | 0.017 | 0.585 | 0.559 |  |  |
|  | PH | <--- | age | | -0.050 | 0.013 | -3.750 | *** |  |  |
|  | PH | <--- | drink | | 0.018 | 0.014 | 1.294 | 0.196 |  |  |
|  | MH | <--- | EC | | 0.002 | 0.015 | 0.145 | 0.885 | 0.305 |  |
|  | MH | <--- | INE | | 0.195 | 0.067 | 2.925 | 0.003 |  |  |
|  | MH | <--- | SS | | -0.670 | 0.082 | -8.197 | *** |  |  |
|  | MH | <--- | PH | | 0.036 | 0.005 | 6.631 | *** |  |  |
|  | MH | <--- | education | | -0.090 | 0.059 | -1.520 | 0.129 |  |  |
|  | MH | <--- | age | | -0.127 | 0.043 | -2.950 | 0.003 |  |  |
|  | MH | <--- | drink | | 0.050 | 0.048 | 1.050 | 0.294 |  |  |
|  | PF | <--- | EC | | -0.171 | 0.102 | -1.678 | 0.093 | 0.211 |  |
|  | PF | <--- | INE | | 3.017 | 0.432 | 6.990 | *** |  |  |
|  | PF | <--- | SS | | -1.813 | 0.376 | -4.822 | *** |  |  |
|  | PF | <--- | education | | -0.021 | 0.396 | -0.053 | 0.958 |  |  |
|  | PF | <--- | age | | 2.848 | 0.271 | 10.522 | *** |  |  |
|  | PF | <--- | drink | | -0.097 | 0.320 | -0.304 | 0.761 |  |  |
| Model fitting index | | fitting index | χ2 /df | | CFI | TLI | NFI | IFI | RFI | RMSEA |
|  |  | standards | <5 | | >0.9 | >0.9 | >0.9 | >0.9 | >0.9 | <0.05 |
|  |  | Model results | 3.264 | | 0.925 | 0.910 | 0.896 | 0.926 | 0.875 | 0.04 |

Note: *** significant at P<0.001.

2、**age+ education+ lifestyle（smoke）**

| **Model** | **Variable relationship** | | | | **Estimate** | **S.E.** | **C.R.** | **P** | **R^2^** |  |
| --- | --- | --- | --- | --- | --- | --- | --- | --- | --- | --- |
| Structural Equation Model | PH | <--- | | EC | 0.009 | .004 | 2.160 | .031 | 0.139 |  |
|  | PH | <--- | | INE | -0.038 | .019 | -2.017 | .044 |  |  |
|  | PH | <--- | | SS | -0.046 | .017 | -2.658 | .008 |  |  |
|  | PH | <--- | | PF | 0.010 | .002 | 5.314 | *** |  |  |
|  | PH | <--- | | education | 0.010 | .017 | .583 | .560 |  |  |
|  | PH | <--- | | age | -0.050 | .013 | -3.753 | *** |  |  |
|  | PH | <--- | | smoke | 0.013 | .013 | .952 | .341 |  |  |
|  | MH | <--- | | EC | 0.001 | 0.015 | 0.043 | 0.966 | 0.305 |  |
|  | MH | <--- | | INE | 0.190 | 0.067 | 2.852 | 0.004 |  |  |
|  | MH | <--- | | SS | -0.666 | 0.081 | -8.202 | *** |  |  |
|  | MH | <--- | | PF | 0.036 | 0.005 | 6.660 | *** |  |  |
|  | MH | <--- | | education | -0.072 | 0.059 | -1.218 | 0.223 |  |  |
|  | MH | <--- | | age | -0.127 | 0.043 | -2.972 | 0.003 |  |  |
|  | MH | <--- | | smoke | -0.061 | 0.046 | -1.328 | 0.184 |  |  |
|  | PF | <--- | | EC | -0.165 | 0.102 | -1.621 | 0.105 | 0.212 |  |
|  | PF | <--- | | INE | 3.026 | 0.431 | 7.018 | *** |  |  |
|  | PF | <--- | | SS | -1.796 | 0.375 | -4.795 | *** |  |  |
|  | PF | <--- | | education | -0.095 | 0.397 | -0.240 | 0.810 |  |  |
|  | PF | <--- | | age | 2.846 | 0.271 | 10.520 | *** |  |  |
|  | PF | <--- | | smoke | 0.348 | 0.311 | 1.121 | 0.262 |  |  |
| Model fitting index | | fitting index | | χ2 /df | CFI | TLI | NFI | IFI | RFI | RMSEA |
|  |  | standards | | <5 | >0.9 | >0.9 | >0.9 | >0.9 | >0.9 | <0.05 |
|  |  | Model results | | 3.29 | 0.924 | 0.909 | 0.896 | 0.925 | 0.874 | 0.04 |

Note: *** significant at P<0.001.

3、**age+ education+ lifestyle（vegetable）**

| **Model** | **Variable relationship** | | | **Estimate** | **S.E.** | **C.R.** | **P** | **R^2^** |  |
| --- | --- | --- | --- | --- | --- | --- | --- | --- | --- |
| Structural Equation Model | PH | <--- | EC | 0.010 | 0.004 | 2.175 | 0.050 | 0.139 |  |
|  | PH | <--- | INC | -0.038 | 0.019 | -2.051 | 0.04 |  |  |
|  | PH | <--- | SS | -0.047 | 0.017 | -2.676 | 0.008 |  |  |
|  | PH | <--- | PF | 0.010 | 0.002 | 5.304 | *** |  |  |
|  | PH | <--- | education | 0.011 | 0.017 | 0.695 | 0.487 |  |  |
|  | PH | <--- | age | -0.049 | 0.013 | -3.716 | *** |  |  |
|  | PH | <--- | vegetable | 0.012 | 0.016 | 0.750 | 0.453 |  |  |
|  | MH | <--- | EC | 0.002 | 0.015 | 0.130 | 0.896 | 0.305 |  |
|  | MH | <--- | INC | 0.190 | 0.067 | 2.859 | 0.004 |  |  |
|  | MH | <--- | SS | -0.668 | 0.082 | -8.192 | *** |  |  |
|  | MH | <--- | PF | 0.036 | 0.005 | 6.656 | *** |  |  |
|  | MH | <--- | education | -0.087 | 0.059 | -1.473 | 0.141 |  |  |
|  | MH | <--- | age | -0.124 | 0.043 | -2.883 | 0.004 |  |  |
|  | MH | <--- | vegetable | 0.072 | 0.058 | 1.242 | 0.214 |  |  |
|  | PF | <--- | EC | -0.173 | 0.102 | -1.696 | 0.090 | 0.212 |  |
|  | PF | <--- | INC | 3.025 | 0.431 | 7.015 | *** |  |  |
|  | PF | <--- | SS | -1.805 | 0.375 | -4.816 | *** |  |  |
|  | PF | <--- | education | -0.015 | 0.394 | -0.039 | 0.969 |  |  |
|  | PF | <--- | age | 2.831 | 0.271 | 10.437 | *** |  |  |
|  | PF | <--- | vegetable | -0.403 | 0.385 | -1.048 | 0.295 |  |  |
| Model fitting index | | fitting index | χ2 /df | CFI | TLI | NFI | IFI | RFI | RMSEA |
|  |  | standards | <5 | >0.9 | >0.9 | >0.9 | >0.9 | >0.9 | <0.05 |
|  |  | Model results | 3.223 | 0.926 | 0.911 | 0.879 | 0.927 | 0.876 | 0.04 |

Note: *** significant at P<0.001.

4、**age+ education+ lifestyle（fruit）**

| **Model** | **Variable relationship** | | | | **Estimate** | **S.E.** | **C.R.** | **P** | **R^2^** |  |
| --- | --- | --- | --- | --- | --- | --- | --- | --- | --- | --- |
| Structural Equation Model | PH | <--- | | EC | 0.009 | 0.004 | 2.136 | 0.033 | 0.138 |  |
|  | PH | <--- | | INC | -0.038 | 0.019 | -2.034 | 0.042 |  |  |
|  | PH | <--- | | SS | -0.046 | 0.017 | -2.673 | 0.008 |  |  |
|  | PH | <--- | | PF | 0.010 | 0.002 | 5.299 | *** |  |  |
|  | PH | <--- | | education | 0.012 | 0.017 | 0.751 | 0.453 |  |  |
|  | PH | <--- | | age | -0.050 | 0.013 | -3.752 | *** |  |  |
|  | PH | <--- | | fruit | -0.003 | 0.011 | -0.294 | 0.769 |  |  |
|  | MH | <--- | | EC | 0.002 | 0.015 | 0.122 | 0.903 | 0.302 |  |
|  | MH | <--- | | INC | 0.192 | 0.067 | 2.880 | 0.004 |  |  |
|  | MH | <--- | | SS | -0.664 | 0.081 | -8.168 | *** |  |  |
|  | MH | <--- | | PF | 0.036 | 0.005 | 6.619 | *** |  |  |
|  | MH | <--- | | education | -0.088 | 0.059 | -1.485 | 0.138 |  |  |
|  | MH | <--- | | age | -0.126 | 0.043 | -2.947 | 0.003 |  |  |
|  | MH | <--- | | fruit | 0.032 | 0.038 | 0.852 | 0.394 |  |  |
|  | PF | <--- | | EC | -0.167 | 0.102 | -1.638 | 0.101 | 0.211 |  |
|  | PF | <--- | | INC | 3.015 | 0.431 | 6.991 | *** |  |  |
|  | PF | <--- | | SS | -1.802 | 0.375 | -4.808 | *** |  |  |
|  | PF | <--- | | education | -0.057 | 0.395 | -0.145 | 0.880 |  |  |
|  | PF | <--- | | age | 2.851 | 0.271 | 10.534 | *** |  |  |
|  | PF | <--- | | fruit | 0.177 | 0.251 | 0.704 | 0.479 |  |  |
| Model fitting index | | fitting index | | χ2 /df | CFI | TLI | NFI | IFI | RFI | RMSEA |
|  |  | standards | | <5 | >0.9 | >0.9 | >0.9 | >0.9 | >0.9 | <0.05 |
|  |  | Model results | | 3.232 | 0.926 | 0.911 | 0.879 | 0.927 | 0.876 | 0.040 |

Note: *** significant at P<0.001.
