# Supplementary material for: Effect of family "upward" intergenerational support on the health of rural elderly in China: Evidence from Chinese Longitudinal Healthy Longevity Survey
Source: PLoS One. 2021 Jun 18;16(6):e0253131. doi: 10.1371/journal.pone.0253131 (PMC8213075; doi:10.1371/journal.pone.0253131)
Supplement: S3 Table — (DOCX) [file pone.0253131.s005.docx]

**Multiple Indicators and Multiple Causes (MIMC)（2014 data）**

**Parameter Estimation Results for Multiple Indicators and Multiple Causes（N=1407）**

| **Model** | **Variable relationship** | | | | | **Estimate** | | **S.E.** | | **C.R.** | | **P** | | **R^2^** | |
| --- | --- | --- | --- | --- | --- | --- | --- | --- | --- | --- | --- | --- | --- | --- | --- |
| Multiple Indicators and Multiple Causes（MIMIC） | PH | <--- | economic support | | | 0.003 | | 0.001 | | 2.073 | | 0.038 | | 0.133 | |
|  | PH | <--- | provide care time | | | 0.007 | | 0.002 | | 3.451 | | *** | |  |  |
|  | PH | <--- | chat | | | -0.054 | | 0.012 | | -4.532 | | *** | |  |  |
|  | PH | <--- | Living standard | | | -0.021 | | 0.009 | | -2.212 | | 0.027 | |  |  |
|  | PH | <--- | PF | | | 0.008 | | 0.002 | | 4.709 | | *** | |  |  |
|  | PH | <--- | education | | | 0.010 | | 0.015 | | 0.680 | | 0.497 | |  |  |
|  | PH | <--- | vegetable | | | 0.011 | | 0.015 | | 0.763 | | 0.445 | |  |  |
|  | MH | <--- | economic support | | | -0.001 | | 0.005 | | -0.163 | | 0.871 | | 0.208 | |
|  | MH | <--- | provide care time | | | 0.009 | | 0.008 | | 1.133 | | 0.257 | |  |  |
|  | MH | <--- | chat | | | 0.011 | | 0.039 | | 0.285 | | 0.776 | |  |  |
|  | MH | <--- | Living standard | | | -0.371 | | 0.040 | | -9.352 | | *** | |  |  |
|  | MH | <--- | PF | | | 0.037 | | 0.005 | | 7.000 | | *** | |  |  |
|  | MH | <--- | education | | | -0.096 | | 0.059 | | -1.619 | | 0.105 | |  |  |
|  | MH | <--- | vegetable | | | 0.079 | | 0.058 | | 1.369 | | 0.171 | |  |  |
|  | HF | <--- | economic support | | | -0.122 | | 0.036 | | -3.361 | | *** | | 0.157 | |
|  | HF | <--- | provide care time | | | 0.419 | | 0.050 | | 8.315 | | *** | |  |  |
|  | HF | <--- | chat | | | 2.647 | | 0.255 | | 10.378 | | *** | |  |  |
|  | HF | <--- | Living standard | | | -1.036 | | 0.228 | | -4.542 | | *** | |  |  |
|  | HF | <--- | education | | | 0.125 | | 0.395 | | 0.315 | | 0.753 | |  |  |
|  | HF | <--- | vegetable | | | -0.491 | | 0.385 | | -1.274 | | 0.203 | |  |  |
| Model fitting index | | fitting index | χ2 /df | | CFI | TLI | | NFI | | IFI | | RFI | | RMSEA | |
|  |  | standards | <5 | | >0.9 | >0.9 | | >0.9 | | >0.9 | | >0.9 | | <0.05 | |
|  |  | Model results | 3.057 | | 0.936 | 0.917 | | 0.909 | | 0.937 | | 0.882 | | 0.038 | |

Note: *** significant at P<0.001.
