# Supplementary material for: Effect of family "upward" intergenerational support on the health of rural elderly in China: Evidence from Chinese Longitudinal Healthy Longevity Survey
Source: PLoS One. 2021 Jun 18;16(6):e0253131. doi: 10.1371/journal.pone.0253131 (PMC8213075; doi:10.1371/journal.pone.0253131)
Supplement: S4 Table — (DOCX) [file pone.0253131.s006.docx]

**Adjust the study sample（2018 data）**

1. **Factor Analysis**

**（1）KMO and Bartlett's Test**

| **KMO and Bartlett's Test** | | |
| --- | --- | --- |
| Kaiser-Meyer-Olkin Measure of Sampling Adequacy. | | 0.706 |
| Bartlett's Test of Sphericity | Approx. Chi-Square | 9893.471 |
|  | df | 136 |
|  | Sig. | 0.000 |

（2）**Total Variance Explained**

| **Total Variance Explained** | | | | | | | | | |
| --- | --- | --- | --- | --- | --- | --- | --- | --- | --- |
| Component | Initial Eigenvalues | | | Extraction Sums of Squared Loadings | | | Rotation Sums of Squared Loadings | | |
|  | Total | % of Variance | Cumulative % | Total | % of Variance | Cumulative % | Total | % of Variance | Cumulative % |
| 1 | 3.192 | 18.779 | 18.779 | 3.192 | 18.779 | 18.779 | 2.489 | 14.640 | 14.640 |
| 2 | 2.045 | 12.032 | 30.811 | 2.045 | 12.032 | 30.811 | 1.915 | 11.263 | 25.902 |
| 3 | 1.716 | 10.094 | 40.905 | 1.716 | 10.094 | 40.905 | 1.744 | 10.261 | 36.163 |
| 4 | 1.389 | 8.172 | 49.076 | 1.389 | 8.172 | 49.076 | 1.717 | 10.099 | 46.262 |
| 5 | 1.196 | 7.037 | 56.113 | 1.196 | 7.037 | 56.113 | 1.505 | 8.855 | 55.117 |
| 6 | 1.161 | 6.828 | 62.941 | 1.161 | 6.828 | 62.941 | 1.330 | 7.824 | 62.941 |
| 7 | 0.917 | 5.396 | 68.337 |  |  |  |  |  |  |
| 8 | 0.822 | 4.837 | 73.174 |  |  |  |  |  |  |
| 9 | 0.762 | 4.480 | 77.654 |  |  |  |  |  |  |
| 10 | 0.693 | 4.075 | 81.729 |  |  |  |  |  |  |
| 11 | 0.641 | 3.772 | 85.501 |  |  |  |  |  |  |
| 12 | 0.556 | 3.268 | 88.770 |  |  |  |  |  |  |
| 13 | 0.516 | 3.034 | 91.804 |  |  |  |  |  |  |
| 14 | 0.487 | 2.866 | 94.669 |  |  |  |  |  |  |
| 15 | 0.373 | 2.195 | 96.865 |  |  |  |  |  |  |
| 16 | 0.347 | 2.040 | 98.905 |  |  |  |  |  |  |
| 17 | 0.186 | 1.095 | 100.000 |  |  |  |  |  |  |
| Extraction Method: Principal Component Analysis. | | | | | | | | | |

（3）**Rotated Component Matrix**

| **Rotated Component Matrix^a^** | | | | | | |
| --- | --- | --- | --- | --- | --- | --- |
|  | Component | | | | | |
|  | 1 | 2 | 3 | 4 | 5 | 6 |
| outpatient | -0.021 | 0.028 | 0.014 | 0.000 | 0.017 | 0.817 |
| Inpatient | -0.014 | 0.053 | 0.062 | 0.041 | -0.075 | 0.802 |
| are you nervous and scared?(b36) | -0.021 | 0.039 | 0.809 | 0.024 | -0.022 | 0.049 |
| do you feel lonely?(b38) | 0.168 | 0.009 | 0.771 | -0.007 | -0.063 | -0.011 |
| do you think that the older you are, the less useful you are, and the hard work(b34) | -0.014 | 0.197 | 0.655 | 0.011 | -0.165 | 0.049 |
| ADL | 0.030 | 0.815 | 0.035 | -0.052 | -0.047 | 0.038 |
| IADL | 0.278 | 0.812 | 0.098 | -0.053 | -0.043 | -0.013 |
| STGN | 0.119 | 0.297 | 0.140 | 0.096 | -0.209 | 0.002 |
| LIAOTIAN1 | 0.843 | 0.235 | 0.016 | 0.033 | 0.014 | -0.027 |
| XINSHI1 | 0.920 | 0.137 | 0.064 | 0.055 | 0.017 | -0.004 |
| KUNNAN1 | 0.872 | 0.089 | 0.063 | 0.086 | 0.011 | -0.016 |
| SJZL | 0.089 | 0.593 | 0.042 | 0.047 | 0.115 | 0.058 |
| is all of the financial support sufficient to pay for daily expenses?(f33) | 0.053 | 0.008 | -0.107 | 0.000 | 0.829 | -0.070 |
| SHSP | 0.006 | -0.011 | -0.094 | 0.078 | 0.844 | 0.008 |
| RZDS | 0.009 | -0.049 | -0.006 | 0.815 | 0.010 | 0.032 |
| NRDS | -0.039 | -0.064 | -0.007 | 0.828 | -0.025 | -0.026 |
| SBDS | 0.194 | 0.137 | 0.044 | 0.574 | 0.072 | 0.039 |
| Extraction Method: Principal Component Analysis.  Rotation Method: Varimax with Kaiser Normalization. | | | | | | |
| a. Rotation converged in 6 iterations. | | | | | | |

1. **Parameter estimation results for Structural equation model （2018data）**

**（1）Structural equation model of the effect of intergenerational support on the health of the elderly in rural areas and its mechanism（2018 data）**

**
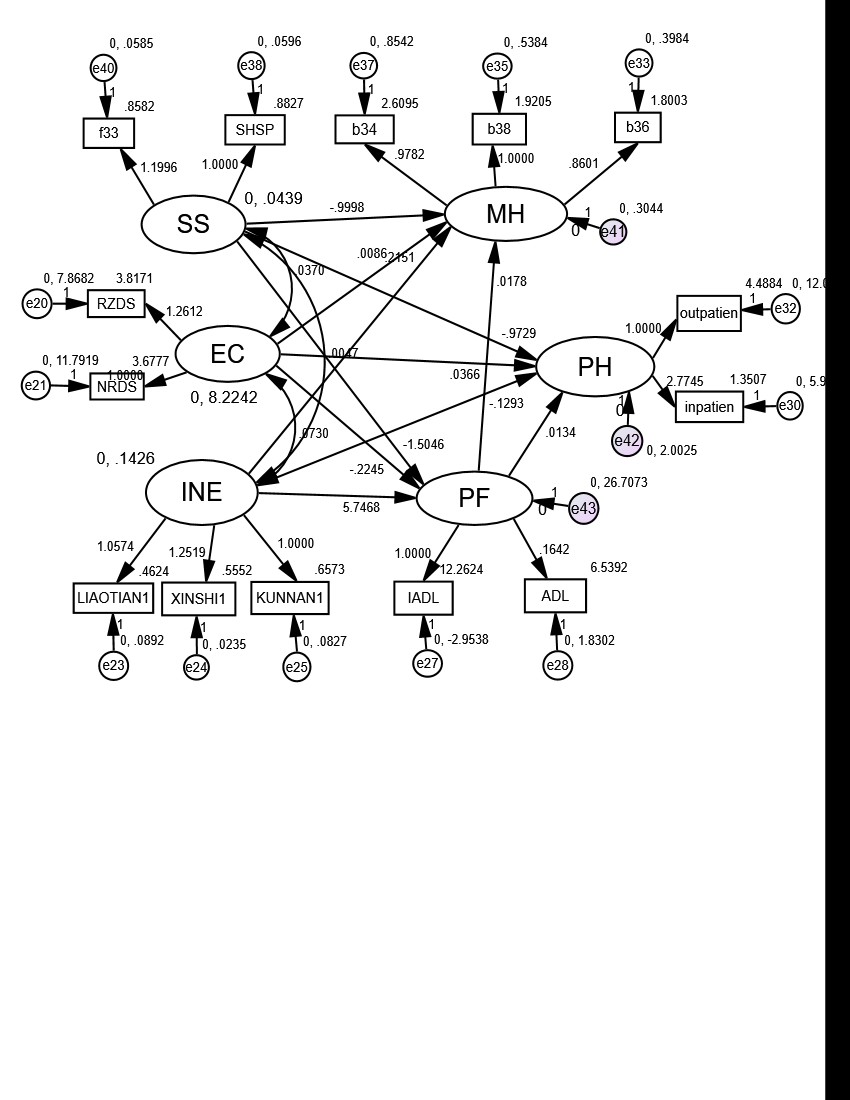
**

**（2）Parameter estimation results for Structural equation model (N = 2489)**

| **Model** | **Variable relationship** | | | **Estimate** | **S.E.** | **C.R.** | **P** | **R^2^** |
| --- | --- | --- | --- | --- | --- | --- | --- | --- |
| Structural Equation Model | PH | <--- | EC | 0.037 | 0.020 | 1.809 | 0.070 | 0.027 |
|  | PH | <--- | INE | -0.129 | 0.119 | -1.084 | 0.278 |  |
|  | PH | <--- | SS | -0.973 | 0.386 | -2.521 | 0.012 |  |
|  | PH | <--- | PF | 0.013 | 0.009 | 1.568 | 0.117 |  |
|  | MH | <--- | EC | 0.009 | 0.006 | 1.379 | 0.168 | 0.178 |
|  | MH | <--- | INE | 0.215 | 0.048 | 4.501 | *** |  |
|  | MH | <--- | SS | -1.000 | 0.102 | -9.833 | *** |  |
|  | MH | <--- | PF | 0.018 | 0.003 | 5.474 | *** |  |
|  | PF | <--- | EC | -0.225 | 0.042 | -5.365 | *** | 0.157 |
|  | PF | <--- | INE | 5.747 | 0.288 | 19.968 | *** |  |
|  | PF | <--- | SS | -1.505 | 0.595 | -2.531 | 0.011 |  |
| Model fitting index | fitting index | χ2 /df | CFI | TLI | NFI | IFI | RFI | RMSEA |
|  | standards | <5 | >0.9 | >0.9 | >0.9 | >0.9 | >0.9 | <0.05 |
|  | Model results | 4.785 | 0.973 | 0.961 | 0.966 | 0.973 | 0.951 | 0.039 |

Note: *** significant at P<0.001.
